# Environmental Determinants of Ferroptosis in Cancer

**DOI:** 10.3390/cancers15153861

**Published:** 2023-07-29

**Authors:** Yasaman Setayeshpour, Yunji Lee, Jen-Tsan Chi

**Affiliations:** 1 Department of Molecular Genetics and Microbiology, Duke University Medical Center, Durham, NC 27708, USA; 2 Department of Cell and Molecular Biology, Duke University Medical Center, Durham, NC 27708, USA; 3 Division of Natural and Applied Sciences, Duke Kunshan University, Kunshan 215316, China; 4 Center for Advanced Genomic Technology, Duke University, Durham, NC 27708, USA

**Keywords:** ferroptosis, cancer, metastasis, TME, lipid peroxidation, iron

## Abstract

**Simple Summary:**

The environment which cancer cells are exposed to can heavily influence the rate of their growth, progression, and metastasis. Many metastasizing cancer cells are vulnerable to a particular type of cell death known as ferroptosis, which is an iron-dependent form of cell death caused by accumulative oxidative stress. Interestingly, many intracellular and extracellular factors can influence ferroptosis and, therefore, dictate the efficiency and route of tumor metastasis. In this review, we will go over key established intracellular factors and highlight major emerging understandings of extracellular factors of ferroptosis in the context of cancer.

**Abstract:**

Given the enormous suffering and death associated with human cancers, there is an urgent need for novel therapeutic approaches to target tumor growth and metastasis. While initial efforts have focused on the dysregulated oncogenic program of cancer cells, recent focus has been on the modulation and targeting of many “cancer-friendly,” non-genetic tumor microenvironmental factors, which support and enable tumor progression and metastasis. Two prominent examples are anti-angiogenesis and immunotherapy that target tumor-supporting vascularization and the immune-suppressive tumor microenvironment (TME), respectively. Lately, there has been significant interest in the therapeutic potential of ferroptosis, a natural tumor suppression mechanism that normally occurs as a result of oxidative stress, iron imbalance, and accumulation of lipid peroxides. While numerous studies have identified various cell intrinsic mechanisms to protect or promote ferroptosis, the role of various TME stress factors are also recently recognized to modulate the tumor cells’ susceptibility to ferroptosis. This review aims to compile and highlight evidence of these factors, how various TME stresses affect ferroptosis, and their implications in various stages of tumor development and expected response to ferroptosis-triggering therapeutics under development. Consequently, understanding ways to enhance ferroptosis sensitivity both intracellularly and in the TME may optimize therapeutic sensitivity to minimize or prevent tumor growth and metastasis.

## 1. Introduction

The tumor microenvironment (TME) is the cellular and chemical environment around a tumor, including the surrounding blood vessels, immune cells, fibroblasts, signaling molecules, and the extracellular matrix with various chemical and physical parameters [1]. The TME is often altered by tumors and can directly influence how tumors grow and spread, as well as how they respond to treatments [1]. For example, tumors often outgrow their blood supply, leading to areas of low oxygen or hypoxia. Hypoxia can make tumor cells more resistant to therapies, particularly radiation therapy, which requires oxygen to produce reactive oxygen species and cause DNA damage [2]. Another example includes the accumulation of lactic acid, which leads to a lower pH (acidic) environment and can limit the effectiveness of some drugs and, hence, promote a more aggressive tumor phenotype [3,4]. In addition, the TME often has a high interstitial fluid pressure and a dense extracellular matrix, which can physically prevent drugs from reaching the tumor cells [1]. Yet another important feature of the TME is the metabolic competition between rapidly proliferating cancer cells and the immune cells, with the cancer cells often outcompeting immune cells for nutrients, thereby suppressing immune function and potentially reducing the efficacy of immunotherapies [1].

Due to these extracellular influences on top of bearing significant genomic instability and a high frequency of somatic mutations, tumor cells bearing certain mutations may have survival or proliferation advantages and become “selected” for while exposed to various TME stresses and become the dominant component of tumors. This phenomenon is known as “somatic evolution” [5]. During somatic evolution, the tumor microenvironmental stresses serve as an important factor in selecting cancer cells, such as hypoxia enriched sites selecting for tumor cells that lack p53 [6,7], and glucose deprived sites selecting for tumor cells that bear Kras mutations [8]. While oncogenic driver mutations in tumor cells are often assumed to confer growth advantages due to proliferation or reduced cell death, many genes noted in the somatic mutations and copy number alterations (CNAs) in, for example, breast cancer may offer survival only under stresses [9]. This was shown by our findings that activating transcription factor 4 (ATF4) amplification and a higher ATF4-driven gene expression program in a subset of breast cancer cells provides a survival advantage under hypoxia and lactic acidosis [10]. These influences of the TME can also promote the development of drug-resistant tumor cells [5] and, therefore, make the TME an important target in cancer therapy. Strategies to normalize the TME, such as improving tumor blood flow [11] or targeting immunosuppressive cells [12], are under active investigation and could potentially enhance the effectiveness of existing cancer therapies. More importantly, more is being discovered about the specific death-inducing stresses within the TME and how they can be subverted by the metastasizing tumor cells [13,14,15]. Understanding this can further facilitate investigation of potential therapeutic approaches.

## 2. Ferroptosis, an Iron-Dependent Form of Regulated Cell Death

As indicated, genomic instability and dysregulated cellular processes due to the high frequency of mutations are one of the main drivers of tumor growth within the TME [5]. Therefore, maintaining genomic stability and homeostasis of major cellular processes is crucial for preventing the transformation of healthy cells into cancer cells [5,16]. Normally, several checkpoints and defense systems are in place to repair and reverse any error or imbalance in key cellular processes; in the case of excessive and irreversible cellular damage, different types of programmed cell deaths can be activated to kill malfunctioning cells as an effective tumor suppression mechanism [16,17]. The best-recognized type of cell death is apoptosis, which is triggered by DNA damage and other stresses to remove damaged cells [17]. It is well established that defects in the process of apoptosis play an important role in the tumor formation and drug resistance of cancer cells [18]. Another important cellular process to maintain is a balanced level of reactive oxygen species (ROS), which are often a product of high efficiency energy generation using oxygen-driven respiration [19,20]. While ROS are byproducts of necessary cellular processes and essential for signaling functions [19], excessive levels can lead to a toxic overaccumulation of oxidized cellular components, such as lipid peroxides [20]. Thus, there is a need for cellular antioxidant defense systems to repair the resulting lipid peroxides. In the case of excessive, irreversible membrane damage and lipid peroxidation, a specific type of iron-dependent programmed cell death called ferroptosis occurs [21,22].

Ferroptosis is a recently recognized form of regulated cell death characterized by iron dependency, lipid peroxidation, mitochondria shrinkage, and membrane content condensation [21]. Ferroptosis was first defined by Dixon et al. (2012) when they were studying the cell death mechanisms by which the small molecule drug erastin kills RAS-mutated cancer cells [23]. It has now been more than a decade since this first publication on ferroptosis, and since then, there have been significant advances in understanding the processes and determinants of ferroptosis, especially at the cellular level [24]. Ferroptosis occurs when cellular ROS generation and resulting lipid peroxidation outweigh cellular neutralizing mechanisms, including various antioxidants and enzymatic activities removing lipid peroxidation. Some of the main producers of ROS are Nicotinamide adenine dinucleotide phosphate (NADPH) oxidases (NOXs), which can generate ROS as a result of electron transfer to oxygen molecules [25]. This results in the accumulation of lipid peroxidation, which would trigger ferroptosis unless lipid peroxidation is neutralized by dedicated lipid repair enzymes and antioxidant generation [24]. 

As we will discuss in this review, due to their altered metabolism and having to pass through various microenvironmental stressors in order to metastasize, cancer cells are especially susceptible to ferroptotic cell death [15]. Interestingly, however, despite being more vulnerable to ferroptosis, many factors can protect cancer cells from ferroptosis and therefore enable the progression of metastasis [15]. Understanding how these mechanisms work and investigating ways to dismantle ferroptosis resistance both at the cellular and extracellular level has the potential to be a powerful therapeutic approach for preventing or minimizing cancer metastasis.

## 3. Genetic Regulators and Protectors of Ferroptosis 

Ferroptosis has garnered a significant amount of attention in cancer studies because many oncogenic mutations and cellular states, while conferring resistance to various therapeutics, strongly promote ferroptosis [14]. Therefore, triggering ferroptosis has been shown to eliminate these ferroptosis-sensitive cancer cells with significant and promising therapeutic potential; however, it is still difficult to predict which tumors are most sensitive and would respond best to ferroptosis-inducing therapeutics [14]. As a result, many functional genomic studies have been employed to identify the genetic determinants of ferroptosis. These studies have revealed the importance of genetic regulation of several processes, such as the mesenchymal state, iron metabolism, lipid reprogramming, and the trans-sulfation pathways in ferroptosis [14]. Below, we will highlight some major intracellular determinants of ferroptosis.

### 3.1. The GPX4-Dependent and -Independent Antioxidant Defense Pathways

First, several major antioxidant defense mechanisms are in place to prevent ferroptosis from occurring. Namely, the lipid repair enzyme glutathione peroxidase 4 (GPX4), is a major intracellular protector against ferroptosis, which uses the antioxidant glutathione (GSH) as a co-factor [26]. GSH, in turn, is generated via the import of cystine through the xCT transporter [26]. Both GPX4 and xCT are downstream targets of the transcription factor erythroid 2-related factor 2 (NRF2), which is a master regulator of related antioxidant proteins [27]. In addition, the recently discovered ferroptosis suppressor protein 1 (FSP1), which is also known as AIFM2, has been shown to mediate a protection mechanism against ferroptosis, as well. FSP1 works in a GPX4-independent manner by reducing ubiquinone (also known as Coenzyme Q_10_) to ubiquinol, which can act as an antioxidant in its reduced form [28]. In addition, dihydroorotate dehydrogenase (DHODH) had previously been thought to protect against ferroptosis by generating orotate from dihydroorotate [29], but recently, Mishima et al. (2023) show that DHODH inhibitors can only sensitize cells against ferroptosis when used in high concentrations, and these high concentrations have been shown to also affect FSP1 expression levels [30]. Hence, further research is needed to clarify the importance of DHODH for ferroptosis. In the context of cancer studies, several drugs exist that can induce ferroptosis via either depleting or inactivating key components of the antioxidant defense mechanism. For example, erastin, sulfasalazine, and sorafenib trigger ferroptosis by inhibiting cystine uptake and depleting GSH levels [21,31]. In contrast, RSL3, ML-210, and JKE-1674 inhibit GPX4 and its ability to neutralize lipid peroxidation [32].

### 3.2. NADPH

Nicotinamide adenine dinucleotide phosphate (NADPH) also plays a crucial role in protecting cells from ferroptosis [22]. NADPH provides the reducing equivalents for antioxidant systems, such as glutathione and thioredoxin, to function effectively [22]. GSH, in particular, is a critical antioxidant in this context. The reduced form of GSH can directly reduce lipid peroxides to non-toxic lipids, but in the process, GSH itself becomes oxidized. This oxidized glutathione (GSSG) can be converted back to its reduced form by the enzyme glutathione reductase, which uses NADPH as a source of reducing power [33]. When NADPH levels are insufficient, the glutathione system cannot effectively reduce lipid peroxides [33]. This can result in an accumulation of lipid peroxides and trigger ferroptosis [22]. So, in summary, NADPH plays a crucial role in preventing ferroptosis by supplying the necessary reducing power to the glutathione system so that it can effectively neutralize lipid peroxides. This helps in maintaining the redox homeostasis in the cell and prevents the buildup of toxic lipid peroxides that can drive ferroptosis [22,33].

### 3.3. DDR2

The elevated deposition of stromal collagen, a factor that promotes metastasis, is facilitated by the collagen receptor, discoidin domain receptor 2 (DDR2) [34,35]. DDR2 is consistently overexpressed in metastatic and recurrent cancer, which contributes to the establishment of fibroblastic phenotypes of cancer cells. Notably, DDR2 is shown to be highly overexpressed in recurrent breast tumors and plays a crucial role in making cells susceptible to ferroptosis [36]. Such ferroptosis regulation is shown to be mediated through the YAP and TAZ, the two transcriptional coactivators in the Hippo tumor suppression pathways [36]. Considering the capacity of YAP/TAZ and DDR2 to boost both ferroptosis and metastasis, instigating ferroptosis emerges as a potentially effective strategy to tackle cancer cells during the earliest phase of metastasis.

### 3.4. p53

The tumor suppressor protein, p53 has long been considered the gatekeeper of genomic DNA integrity and is lost or mutated in about half of human cancers [37]. p53 is responsible for activating cell death in response to various DNA damage and compromised genomic integrity. The p53-mediated cell death upon DNA damage was thought to occur via apoptosis [37]. Interestingly, however, p53 has also been shown to regulate ferroptosis with both inhibitory and promotive effects depending on the specific biological context [38,39,40,41,42,43,44,45,46]. To elaborate, whether p53 promotes ferroptosis or protects against it seems to heavily depend on specific p53 mutations, especially in the context of cancer [38,42,43,44]. Additionally, ferroptosis promoting effects are usually in a transcription- [40,41] or epigenetic-dependent manner [39] while wildtype p53’s ferroptosis inhibitory effects are usually transcription-independent and are specific to certain cancer cell types, such as colorectal cancer [45].

For example, a mutant form of p53 that can no longer promote cell-cycle arrest, cell senescence and apoptosis can instead promote ferroptosis by suppressing the levels of *SLC7A11* mRNA (which encodes for an xCT subunit) [38]. However, one study shows that both wildtype and mutant p53 can regulate SLC7A11 expression levels via negative regulation of histone H2Bub1 levels, which is responsible for downstream upregulation of *SLC7A11* [39]. Additionally, via its repression of *SLC7A11*, p53 can activate the function of arachidonate 12-lipooxygenase (ALOX12), which has recently been identified as a positive regulator of ferroptosis [40]. An increase in the p53 transcription target, spermidine/spermine N1-acetyltransferase 1 (SAT1), can also promote ferroptosis; SAT1 is crucial for polyamine catabolism and can trigger ferroptosis by augmenting lipid peroxidation in response to ROS [41].

On the other hand, a missense mutant version of p53 in breast cancer cells can inhibit ferroptosis by interacting with the transcription factor NRF2, the master regulator of the antioxidant response; this interaction causes the selective activation and repression of NRF2 target genes and promotes survival of breast cancer cells in an oxidative stress inducing microenvironment, which would otherwise be deadly to these cells [42]. Furthermore, ferroptosis is impaired in cells expressing an African-centric single nucleotide polymorphism (SNP) on codon 47 of p53 (Pro47Ser, rs1800371, G/A) due to higher levels of Coenzyme A (CoA) and GSH [43]. Intriguingly, while P47S p53 variant cells exhibit ferroptosis resistance, presence of this variant also leads to the differentiation of anti-inflammatory macrophages and, hence, better protection against the malarial toxin hemozoin [44]. Other examples of p53 inhibiting ferroptosis can be observed specifically in colorectal cancer cells, in which p53 directly regulates the membrane location of dipeptidyl-peptidase-4 (DPP4) [45]. And yet another study shows that forced expression of wildtype p53 coupled with treatment with the MDM2 inhibitor, nutlin-3, consistently inhibits ferroptosis via induction of a canonical p21 pathway that enhances cystine import and GSH accumulation [46].

As evident by the compiled literature, p53 has a complex and a context-dependent role in regulating ferroptosis. Hence, although many studies have focused on its effects on ferroptosis, further investigation will undoubtedly elucidate additional mechanisms and help clarify its significance for ferroptotic cell death.

### 3.5. SREBP1

Sterol regulatory element-binding protein 1 (SREBP1) is a transcription factor that regulates lipid metabolism and is involved in the biosynthesis of fatty acids, triglycerides, and phospholipids [47]. SREBP1 can regulate ferroptosis by affecting the level of lipid biosynthesis and desaturation since ferroptosis involves the peroxidation of polyunsaturated fatty acids (PUFAs) that leads to cell death [47,48]. SREBP1 could potentially affect the cell’s vulnerability to ferroptosis by influencing the composition of cellular lipids. For example, oncogenic activation of the PI3K-AKT-mTOR signaling promotes oncogenesis by suppressing ferroptosis via the sterol regulatory element-binding protein-mediated lipogenesis, and inhibition of this pathway promotes ferroptosis efficacy [49]. In another study of circulating melanoma cells, transcriptome analysis revealed that an upregulation of SREBP2 directly induces transcription of the iron carrier transferrin (TF), and causes a reduction in intracellular iron pools, ROS, and lipid peroxidation, thereby providing ferroptosis resistance to enable survival in the blood [50].

SREBP1 may also influence the expression of lipid desaturases, such as stearoyl-CoA desaturase (SCD1) [48]. These enzymes introduce double bonds into fatty acid chains, affecting their degree of unsaturation [51]. As lipid peroxidation in ferroptosis preferentially targets polyunsaturated fatty acids, the regulation of these desaturases can affect ferroptosis sensitivity [52]. It is important to note that while these roles suggest a potential connection between SREBP1 and ferroptosis, more research is needed to fully understand the exact mechanisms and to determine whether modulating SREBP1 activity could serve as a therapeutic strategy for diseases where ferroptosis is implicated.

## 4. Microenvironmental Regulators and Protectors of Ferroptosis

As explained, many genetic determinants have been identified for ferroptosis, but less is known about the role of non-genetic factors, understanding of which is critical for utilizing ferroptosis as a therapeutic approach in cancer. Intriguingly, while there are several microenvironmental inducers of ferroptosis in the body, there seem to be just as many protectors against ferroptosis, especially during various key steps of the metastatic cascade of cancer cells [53]. More than 90% of all cancer-related deaths are caused by metastasis, which is the spreading of cancer cells from the primary tumor to other body sites [54,55,56]. Hence, understanding how to dismantle this process is critical for developing effective cancer treatments to improve clinical outcomes.

Metastasis is not an easy process; to establish metastatic tumors, tumor cells need to go through multiple stress-inducing steps [53]. The metastatic cascade initiates with the progressive growth of tumor cells, followed by the local invasion of surrounding tissues, which involves loss of cellular contact and detachment from the extracellular matrix. Then, tumor cells will spread to existing or newly generated blood or lymphatic vessels, where they need to survive long enough during vasculature to become trapped in the vascular walls of distant tissues in order to extravasate. Finally, if the microenvironment in these tissues allows, cancer cells will egress and survive to proliferate, colonize, and form a metastatic tumor in the target organs [53,57,58]. As we will discuss below, these stress-inducing steps make metastasizing cancer cells particularly susceptible to ferroptosis, yet tumor cells still metastasize due to several protective elements along the way, which indicates that effective metastasis may be enabled by various environmental determinants of ferroptosis.

### 4.1. Cellular Contact

One non-genetic factor that affects ferroptosis is cell density and cellular contact. Recently, we and others observed that sensitivity to ferroptosis of various cancer cells is highly influenced by cell contact and cellular density/confluency [59,60,61,62]. While cancer cells grown at low density were highly sensitive to ferroptosis, they became resistant to ferroptosis when confluent or when grown in three-dimensional (3D) spheres [63,64,65]. As high cell density and cellular contact activate Hippo pathways and suppress two main effectors, Yes-associated protein 1 (YAP) and transcriptional coactivator with PDZ-binding motif (TAZ), we evaluated their role in such density-dependent ferroptosis. We showed that TAZ, instead of YAP, was abundantly expressed in renal and ovarian cancer cells and underwent density-dependent nuclear translocation when placed at low density. Such TAZ activation is responsible for enhanced ferroptosis sensitivity at low cell density since *TAZ* removal rendered cells resistant to ferroptosis, while overexpression of a constitutively active form of TAZ, TAZS89A, enhanced ferroptosis [59]. Consistent with our finding, another group identified that high cell density-enhanced cellular contacts suppressed ferroptosis through inhibiting YAP [65]. Similarly, we have found similar cell density-dependent ferroptosis in breast cancer via the regulation of YAP, another Hippo co-regulator [66]. Similar regulation of ferroptosis by YAP and cell density has been noted in the hepatic stellate cells that play a role in liver cirrhosis [67,68]. Collectively, these studies have shown that the Hippo pathway effectors YAP/TAZ are important determinants of ferroptosis and that cellular contact can affect their influence on the sensitivity to ferroptosis.

### 4.2. Epithelial-Mesenchymal Transition (EMT)

The epithelial-mesenchymal transition (EMT) is when epithelial cancer cells transition into a “mesenchymal” state, which is associated with migratory and invasive phenotypes and is an essential first step of the metastatic cascade process [58]. Interestingly, while EMT confers resistance to diverse therapeutics across various cancer types, this state often promotes ferroptosis [69]. Several genes associated with EMT regulate ferroptosis. Namely, the downregulation of Cadherin 1 (*CDH1*) and upregulation of zinc finger E-box-binding homeodomain 1 (*ZEB1*), which are characteristics of the EMT state, were shown to enhance ferroptosis [70]. Consistently, O-GlcNAcylation of ZEB1-induced mesenchymal state cancer cells exhibit vulnerability to ferroptosis [71], and mesenchymal state-derived sarcoma cells, such as HT1080, is a model cell line for ferroptosis study because of its high sensitivity to ferroptosis-inducing compounds, such as erastin and RSL3 [22]. Notably, the influence of YAP/TAZ and cellular contact on sensitivity to ferroptosis may explain why EMT promotes ferroptosis, as the state is usually followed by loss of cellular contact. It may also explain why cancer cells usually detach and circulate in clumps as cell clusters [72], as the increased cell density that spheroid formation provides has been shown to protect against ferroptosis [63,64,65,72]. Furthermore, some cancer cells have epithelial-mesenchymal plasticity (EMP), which allows them to transition between the two cellular states instead of being fixed to one [73]. EMP may be another factor that can provide ferroptosis resistance [74], though more research needs to be done on it.

### 4.3. Mono- and Polyunsaturated Fatty Acids

After local invasion and dissemination from the extracellular matrix, circulating tumor cells (CTCs) travel via either the vasculature or the lymphatic system, through which they are exposed to various environmental factors during their travel. In a landmark study, Ubellacker et al. (2020) showed that metastasizing melanoma cells are protected from ferroptosis via oleic acid, which is a type of monounsaturated fatty acid present in the lymph [74]. Monounsaturated fatty acids (MUFAs), as well as polyunsaturated fatty acids (PUFAs) are some other non-genetic determinants of ferroptosis [75,76]. Several studies show that MUFAs, such as oleic acid, make cells less susceptible to and protected against ferroptosis [75,76]. In contrast, due to their multiple double bonds, PUFAs, such as linoleic acid, are great substrates for lipid peroxidation and extremely prone to peroxidation. Hence, their increased presence in the cells makes cancer cells more susceptible to ferroptotic cell death due to more available preferred sites for peroxidation [52].

Mechanistically, both MUFAs and PUFAs can either be de novo synthesized via proteins, such as the fatty acid synthase (FASN) [77] and SCD [51], or they can be taken up by the cell from the extracellular environment. These long chain fatty acids can be imported through passive diffusion or through transmembrane proteins, such as CD36, or fatty acid binding proteins (FABPs) [78,79]. PUFAs can promote ferroptosis via their activation by the acid-CoA synthase long-chain family member 4 (ACSL4) and lysophosphatidylcholine acyltransferase 3 (LPCAT3), both of which have been shown to mediate ferroptosis via incorporating PUFAs into the plasma membrane [52]. On the other hand, MUFAs can inhibit ferroptosis by their activation via the acid-CoA synthase long-chain family member 3 (ACSL3) and their incorporation into the plasma membrane, which blocks the membrane damage characteristic to ferroptosis [76]. Additionally, the phospholipid-modifying enzymes MBOAT1 and MBOAT2 preferentially use MUFAs as substrates to remodel the plasma membrane and, therefore, provide protection against ferroptosis. MBOAT1/2 can be transcriptionally upregulated via the sex hormone receptors, estrogen receptor (ER) and androgen receptor (AR) [80]. This makes the effect of unsaturated fatty acids on ferroptosis especially relevant in cancers such as ER+ breast cancer or AR+ prostate cancer. Indeed, Liang et al. (2023) shows that when ER or AR antagonists were used with a combination of ferroptosis induction in ER+ breast cancer cells or AR+ prostate cancer cells, they became especially sensitized to the treatment [80]. Therefore, whether CTCs are exposed to PUFAs or MUFAs and the type of cancer cells may be paramount to the CTCs’ fate and whether they are resistant or susceptible to ferroptosis and, hence, likely to effectively metastasize or not.

### 4.4. Adjacent Cells and Exosomes

Aligning with the influence of exogenous fatty acids on CTCs’ resistance or susceptibility to ferroptosis, neighboring adipocytes and adipose-derived exosomes make up another part of the non-genetic determinants of ferroptosis [81,82]. In colorectal cancer, adipose-derived exosomes provide cancer cells’ resistance to ferroptosis via promoting the inhibition of ZEB1 and upregulation of antioxidant genes encoding for GPX4 and xCT, which consequently leads to decreased PUFA and lipid ROS levels [81]. Additionally, in triple-negative breast cancer, co-culturing adipocytes with breast cancer cells protected them from ferroptotic cell death via adipocyte secretion of oleic acid [82].

In addition to adipocytes, cancer-associated fibroblasts (CAFs) are another group that can influence ferroptosis. CAFs are activated fibroblasts that secrete various active factors to regulate tumor development, progression, and treatment response [83]. Recently, CAFs have been found to regulate ferroptosis by providing bioavailable iron to prostate cancer cells and limiting autophagy therapies. Importantly, a low-iron diet was able to reduce this CAF-pancreatic cancer interaction and resensitize the cancer cells to autophagy-based therapeutics [84].

### 4.5. Bioenergetic Depletions

While exposure to different biocomponents can determine resistance or susceptibility to ferroptosis, limited or lack of certain elements, especially nutrients, can also heavily influence the triggering of ferroptosis. Because cancer cells are commonly metabolically altered, they can be especially vulnerable to the deprivation of certain amino acids. We and others have shown that multiple cancer cells, including breast, ovarian, renal, and pancreatic cancer cells, are especially susceptible to cysteine deprivation and undergo cell death upon cystine deprivation [69,85,86,87]. Extracellular cysteine is transported through the xCT transporter, which is then used in the production of the antioxidant GSH, an essential factor for GPX4 [26]. Hence, a depletion in cysteine levels impacts the availability of antioxidants and makes cells much more vulnerable to ferroptosis. Other than GSH, cystine deprivation in pancreatic cells was also found to deplete Coenzyme A (CoA) levels, which is an additional source of metabolites that can protect cells against ferroptosis [87]. In addition to cysteine deprivation, other types of amino acid starvation of cancer cells also impact their sensitivity to ferroptosis. One study shows that pancreatic cancer cells that have been “pseudo-staved” via exposure to mTOR inhibitors and cultured in media lacking FBS, L-glutamine, L-lysine, and L-arginine are sensitive to ferroptosis [88].

Glucose starvation is more complicated; there are conflicting reports of glucose starvation both inhibiting and promoting ferroptosis in different contexts based on distinct cancer cell types via various mechanisms. It has been shown that pancreatic carcinoma cells that were treated with high glucose-containing media are sensitive to ferroptosis due to PDH-mediated pyruvate oxidation. When treated with media lacking glucose, pyruvate dehydrogenase kinase (PDK4), which is a key enzyme in glucose metabolism, becomes activated and can repress pyruvate oxidation, thereby blocking ferroptosis [89]. In renal cell carcinoma, however, glucose deprivation significantly enhances ferroptosis by regulating GPX4 levels. In response to glucose deprivation, the activation of the AMP-activated protein kinase (AMPK), which is a key cellular energy sensor, was shown to significantly reduce GPX4 protein levels via the activation of the p53 gene. GPX4 overexpression or silencing AMPK rescued these cells against ferroptosis [90]. 

### 4.6. Lactate

Microenvironmental levels of lactate can also dictate cancer cells sensitivity to ferroptosis. Lactate-rich liver cancer cells have shown increased resistance to ferroptosis [91]. Mechanistically, lactate uptake through the monocarboxylate transporter 1 (MCT1) leads to downstream activation of stearoyl-coenzyme A (CoA) desaturase-1 (*SCD1*), which is responsible for synthesizing MUFAs [91]. As mentioned, MUFAs are protective against ferroptosis, and their increase results in ferroptosis resistance [75,76]. In non-small cell lung cancer (NSCLC), lactate production was shown to confer ferroptosis resistance via upregulation of GPX4. Mechanistically, GPX4 ubiquitination is attenuated after lactate increase due to the activation of the p38-SGK1 pathway [92].

### 4.7. Hypoxia and Acidity

For cancer cells to effectively settle and colonize in a secondary site, the site must be compromised of certain favorable conditions for the cancer cells. Hypoxia and weak acidity levels are two such features of the tumor microenvironment (TME) [1]. Under normal conditions, hypoxia-inducible factors (HIFs) are tagged for proteasomal degradation, but under hypoxic conditions, HIF-1α and HIF-2α activate and accumulate, which, in turn, cause the transcription of genes involved in the hypoxia response and adaptability [93]. The upregulation of HIF-1α and HIF-2α is observed in many cancer types, and, generally, tumor hypoxia is strongly associated with increased malignancy, poor prognosis, and resistance to various treatment strategies [93,94]. Interestingly, hypoxia has also been shown to promote ferroptosis resistance in hepatocellular carcinoma cells [95]. In HCC cells, hypoxia blocks ferroptosis via HIF-1α-mediated repression of Methyltransferase-like 14 (*METTL14*) and its target gene *SLC7A11* (encoding xCT) [95]. Tumor hypoxia and activation of HIF-1α may also block ferroptosis by reducing PUFA via increased intracellular lipid droplet storage [96].

In addition, HIFs can enhance the performance of the cell’s antioxidant defense system, including GSH and NADPH. Mechanistically, HIF-1 plays a pivotal role in boosting GSH production by elevating the expression of *SLC7A11* and *GCLM*. The increased expression of *SLC7A11*-encoded xCT would consequently increase the cystine import to increase the substrate available for GSH synthesis [97]. HIF-1 also induces the expression *GCLM* that encodes for the glutamate-cysteine ligase modifier subunit, an important component of the γ-glutamylcysteine synthetase enzyme responsible for the initial stage of GSH synthesis by regulating the reaction rate. Together, hypoxia and HIF-1 increase the levels of GSH that can help protect against ferroptosis [97]. Additionally, as mentioned, NADPH is essential for the regeneration of GSH [22] and is found to be depleted during ferroptosis [98]. HIF-1 has been demonstrated to enhance NADPH generation by induction of SHMT2 [99] and PHGDH [100], which produce NADPH through serine metabolism. Consequently, this promotes the preservation of a balanced redox state within the mitochondria under conditions of low oxygen.

While hypoxia and oxidative stresses generally provide favorable conditions for tumor growth, the resulting tumor acidity of the TME can sensitize cancer cells to ferroptosis [101]. In other words, cancer cells’ reliance on hypoxic conditions to survive can be utilized for specific targeting and sensitizing. One recent study has shown that increasing the acidity of the TME via biodegradable nanoparticles can regulate tumor growth via a synergistic induction of ferroptosis, apoptosis, and anti-angiogenesis [102]. This study developed a biodegradable theranostics platform to deliver tamoxifen and glucose oxidase to the tumor site, which resulted in increased aerobic glucose consumption and hypoxic glycolysis in cancer cells, and, in turn, led to significantly increased levels of acidity and overproduction of H_2_O_2_ in the TME [102]; subsequently, this led to tumor cell death via ferroptosis and apoptosis. The study is one example of how knowledge about the non-genetic determinants of ferroptosis can aid in developing anticancer strategies.

### 4.8. Immune Cells

Similar to other types of programmed cell deaths, cells dying via ferroptosis can be recognized via the innate immune system [103]. Usually, dying cells release danger-associated molecular patterns (DAMPs), which can be recognized by pattern-recognition receptors and promote inflammation and the activation of the innate immune system [103]. Along with their production of lipid peroxides, DAMPs like ATP and high-mobility group 1 (HMGB1) are shown to be released by ferroptotic cancer cells [104,105]. Interestingly, HMGB1 has been reported to also regulate ferroptosis; in one study with NRAS-mutant leukemia cells, it was shown that HMGB1-deficient cells show decreased ferroptotic cell death. Mechanistically, the knockdown of *HMGB1* resulted in a significant decrease in c-Jun N-terminal kinase (JNK) and p38 phosphorylation, which, in turn, caused a decrease in TfR1, one of the transmembrane proteins involved in cellular iron uptake. The resulting decrease in iron uptake then causes a decrease in ROS production and, hence, ferroptotic cell death [106]. Hence, varying levels of DAMP expression in cancer cells, especially ones with RAS mutations, which account for 33% of all cancers [106], could influence the frequency of immunogenic ferroptosis and the resulting immune response. Thus, manipulating DAMP levels can be used as an anticancer strategy to enhance the therapeutic benefits of ferroptosis [107]. For example, Hayashi et al. (2020) report that Prostaglandin E_2_ release as a result of chemotherapy with Gemcitabine shows DAMP inhibitory activity [107]. Based on the HMGB1 study with leukemia cells [106], this suggests cells treated with Gemcitabine would have a reduced rate of ferroptotic cell death. One potential future approach could be combining a ferroptosis-inducing agent along with these DAMP inhibitory drugs to ensure ferroptosis sensitivity. Hayashi and colleagues discuss a similar approach and suggest the design of a balanced therapy of immunostimulatory and inhibitory DAMPs for cancer treatment [107].

### 4.9. Broader Influences 

Other than specific microenvironments within the body, more research is being done on the broader influences of metastasis, especially in the context of ferroptotic cell death. Dietary habits seem to be a broad influence [81,108,109]. A study by Zhang et al. (2022) shows that patients with advanced colorectal cancer who also had a high body fat ratio were more resistant to treatment with oxaliplatin due to adipocyte-derived exosomes, making CRC cells less susceptible to ferroptotic cell death [81]. Meanwhile, ingestion of PUFAs induces ferroptosis in *C. elegans* and cancer cells [108], as well as a new study showing that a ketogenic diet in mice can promote tumor ferroptosis [109].

## 5. Conclusions

**Figure 1:**
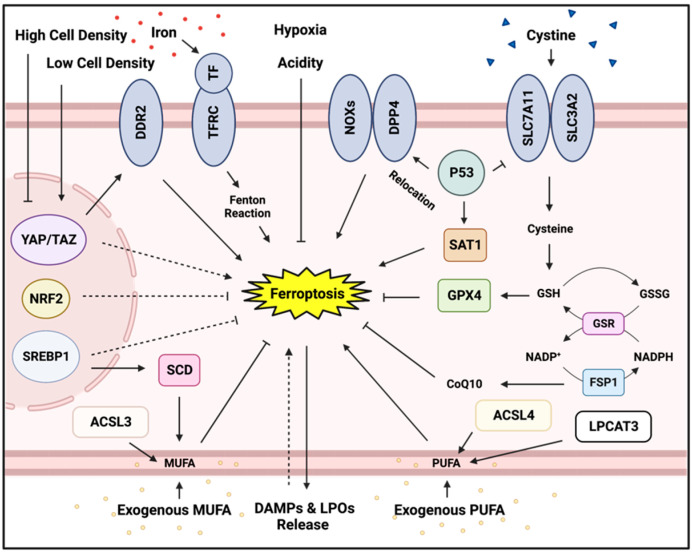
Determinants of ferroptosis. Depiction of key intracellular and extracellular factors that influence the occurrence of ferroptosis. Intracellular factors, such as monounsaturated fatty acid (MUFA) generation, as well as key players in the antioxidant defense system, such as Nrf2, GPX4, FSP1, and the cystine transporter xCT inhibit ferroptosis while iron-dependent Fenton reactions and polyunsaturated fatty acid (PUFA) generation can promote ferroptosis. Extracellularly, cellular contact, bioenergetic availability of amino acids, such as cystine, and the presence of exogenous MUFAs and PUFAs are some of the key factors which influence ferroptosis. Ferroptotic cell death itself can influence the immune cell response via the release of DAMPs and LPOs (lipid peroxides), with some DAMPs also regulating ferroptotic cell death. Schematic was created with BioRender.

Evidence is accumulating that disseminated cancer cells are especially susceptible to ferroptosis, a novel form of regulated cell death, which is distinct from apoptosis and necrosis in many aspects, including the triggering insult, cellular protection mechanisms, metabolic regulation, and biological contexts [21,22,23,24]. Importantly, ferroptosis has been recognized as an important tumor suppressor mechanism with significant therapeutic potential for human cancer [24]. Interestingly, we found that most ovarian cancer cells are especially extremely vulnerable to ferroptosis [85]. Other than the intrinsic ferroptosis-tendency of cancer cells, genetic alternations, including p53 loss and YAP/TAZ activation, also promote ferroptosis. Importantly, many metastasis-associated processes are also associated with ferroptosis, including loss of cell contact, EMT, and matrix detachment, and circulating via potentially hostile microenvironments with an excessive number of ferroptosis-enhancing elements, such as PUFAs and iron. However, as we have highlighted, there are many protective elements against ferroptosis specifically, which may explain why metastasizing cancer cells, which are highly sensitive to ferroptosis, can survive the metastatic process. For example, an important recent study showed that tumor cells in the bloodstream die by ferroptosis but can be rescued from this fate via the MUFA, oleic acid if traveling through the lymph [75]. Other ferroptosis-protecting factors include clumping for increased cellular contact, and influence of adjacent adipocytes and CAFs among other things [64,65,66,67,68,69,70,71,72,73,74,75,76,77,78,79,80,81,82,83,84,85,86,87,88,89,90,91,92,93,94,95,96,97,98,99,100,101,102,103,104,105,106,107,108,109]. Key intracellular and extracellular effects on ferroptosis are illustrated in Figure 1.

**Figure 2:**
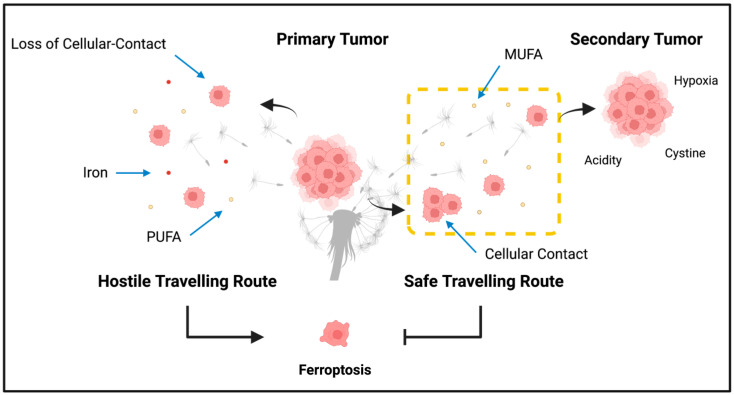
The modified seed and soil model. The classic seed and soil model emphasizes on the importance of favorable conditions in secondary sites for metastasizing cancer cells to settle and colonize in, but the traveling conditions of metastatic cells may be just as important. Metastasizing cancer cells’ sensitivity to ferroptosis makes them extremely vulnerable to the metastasizing process, especially due to the metastasizing steps being associated with promoting ferroptosis and the circulating cancer cells having to travel through potentially hostile microenvironments. However, several ferroptosis-protecting factors are present, which enable the safe traveling of metastasizing cancer cells to a secondary tumor site. Schematic was created with BioRender.

Taken together, it can be concluded that effective metastasis may rely on ferroptosis resistance, and disabling this resistance has an incredibly promising therapeutic potential. The classic “seed and soil” model in cancer studies has been long established [110]. The model emphasizes that metastasis of a tumor is not a “random” occurrence but rather depends on specific, favorable conditions of the secondary site—the “soil”—for the CTCs—the “seeds”—to settle in and colonize [110]. We propose a modified “seed and soil” model, specifically in the context of cancer and ferroptotic cell death, in which the traveling route of these “seeds” is just as important as the colonizing sites of metastasis (Figure 2). It is absolutely critical to consider all potential regulators and protectors of ferroptosis at every step of the metastasis process.

## 6. Future Perspectives

Since ferroptosis was first defined a decade ago, there has been tremendous progress in the understanding of the cell-intrinsic and environmental determinants of ferroptosis, as summarized in several recent outstanding reviews [14,24,111,112,113]. As we embark on the second decade of ferroptosis, we can expect that we will continue to make tremendous progress in the therapeutic application of triggering ferroptosis to prevent tumor progression and optimize therapeutic efficacy for cancer. To accomplish such objectives, it will be critical to understand the environmental determinants to enhance the in vivo efficacy of using ferroptosis and rationalize its combination in therapeutics with other existing anticancer drugs. For example, discovering that ferroptosis resistance is associated with cellular contacts [59,60,61,62,63,64,65,66,67,68,69,70,71,72,73,74] suggests that ferroptosis may be most relevant for the cells which have undergone EMT and lost cellular contacts in the late stage of tumors. Since these tumors are also less likely to respond to conventional chemotherapeutics and ionizing radiation [1], ferroptosis-based therapies may fill this gap that is not well-addressed by current therapeutic approaches. In addition, we can elucidate the different mechanisms of ferroptosis protection and target such mechanisms to resensitize ferroptosis for a therapeutic gain. For example, given the important role of MUFAs in protecting cells against ferroptosis and the required roles of SCD1 and ASCL3 to generate and incorporate MUFA [75,76], targeting SCD1 and ASCL3, together with ferroptosis-inducing agents, may provide a significant therapeutic window to selectively eliminate cancer cells whose survival depends on the availability of MUFAs to survive.

As highlighted in this review, other environmental factors, such as ECM signaling, cell detachment, hypoxia, glycolysis, and acidity, can also heavily regulate ferroptosis [68,69,70,71,72,73,74,75,76,77,78,79,80,81,82,83,84,85,86,87,88,89,90,91,92,93,94]. Therefore, a complete understanding of the environmental determinants of ferroptosis will help overcome the effect of ferroptosis protective factors while enhancing the effect of ferroptosis promoting factors to best optimize ferroptosis-based treatments. However, given the expected spatial heterogeneity of the various cellular, chemical, and physical determinants in different regions of the solid tumors, it is important to take into account a potentially significant level of variation in the response to ferroptosis among different regions of solid tumors. There are several options to address this limitation. First, it is interesting to note that ferroptosis can propagate via adjacent cells, similar to wildfire [114,115]. While the mechanism of such paracrine propagation remains unknown, it is likely that the efficacy of ferroptosis can extend to adjacent cells. Next, since ferroptosis is most likely to be used in a combination therapeutics setting, we can take advantage of such clinical contexts. One study we highlighted in Section 4.7 has already shown the promise of using ferroptosis in the context of combination therapeutics [102]. Other examples include combination of immunotherapies that may enable ferroptosis-enabled CTL to attack tumor cells that are not sensitive to ferroptosis. Similarly, combining ionization radiation with ferroptosis may expand the efficacy beyond the cells undergoing ferroptotic death [116].

It is also important to note that ferroptosis sensitivity has been associated with drug resistance and reported in multiple settings [36,117,118]. Therefore, it is possible to strategically combine these traditional therapeutics with ferroptosis-inducing agents to target different phases or different regions of the tumors. For example, anti-angiogenesis and ionization radiation treatments, administered in sequence or simultaneously, may significantly impact the TME and affect the response to ferroptosis treatments. Similarly, the immune TME may be remodeled and shaped by immunotherapy, which may also impact the ferroptosis response. In future translation applications in clinical settings, it will be important to match individual tumors with ferroptosis-inducing agents and combine with already existing treatments for particular tumor types. For example, tumors with oncogenic mutations that promote ferroptosis may benefit from such treatments. To elaborate, during tumor progression, it is likely that the induction of ferroptosis may be of particular value when used on the disseminated tumor cells that have become resistant to traditional therapeutics but are in a ferroptosis-promoting state. On the other hand, while ferroptosis has been shown to synergize with radiotherapy and immunotherapy, several therapeutic approaches that target the Hippo pathway [60] and DDR2 [36] may limit ferroptosis sensitivity. Therefore, carefully evaluating combination therapeutics is essential for optimal clinical benefits. Finally, not all ferroptosis-inducing agents are equal, especially in relation to what they target. While certain regulators affect xCT and GPX4 inhibitors, others only affect GPX4 inhibitors [119]. This makes the development of FSP1 inhibitors (which target a GPX4-independent pathway) highly relevant in the clinical setting [28,120]. Therefore, much work remains to be done to tailor and optimize the ferroptosis-inducing therapeutic during the current clinical management regimes in order to fully realize the therapeutic potential for individual patients with cancer.

## Data Availability

Not applicable.
